# 261. COVID-19 and Diabetes Mellitus: Glycemic Control and Risk Factors for Mortality, experience from Pakistan.

**DOI:** 10.1093/ofid/ofac492.339

**Published:** 2022-12-15

**Authors:** Asma Nasim, Sunil Kumar

**Affiliations:** Sindh Institute of Urology and Transplantation, Karachi, Sindh, Pakistan; Sindh Institute of Urology and Transplantation, Karachi, Sindh, Pakistan

## Abstract

**Background:**

Diabetes mellitus (DM) is a chronic disease characterized by glucose dysregulation by insulin deficiency or resistance. DM is one of the risk factors and found to be associated with high mortality in patients with corona virus disease-19 (Covid-19). There are challenges in managing poorly controlled DM among Covid-19 patients. Our aim is to highlight the effect of Covid-19 and DM on each other by studying glycemic control and risk factors for mortality.

**Figure:**
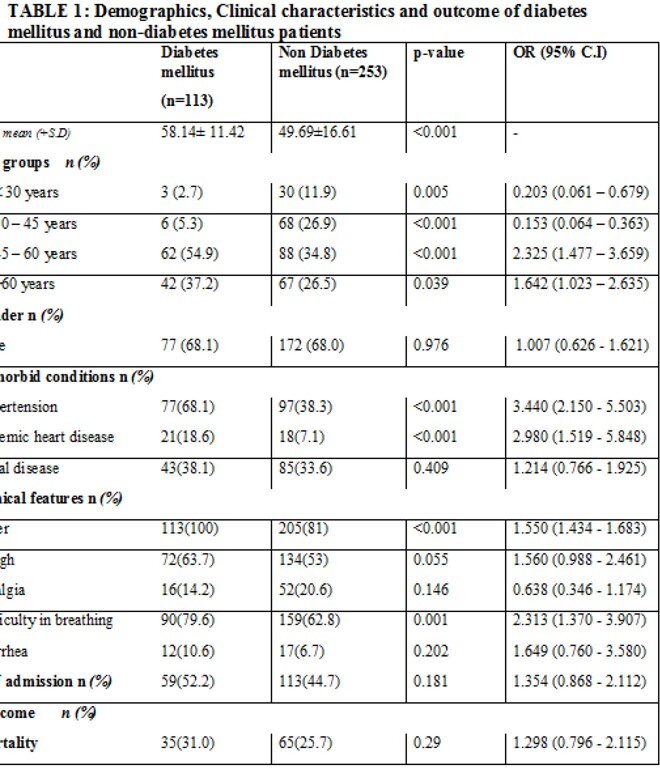

**Figure:**
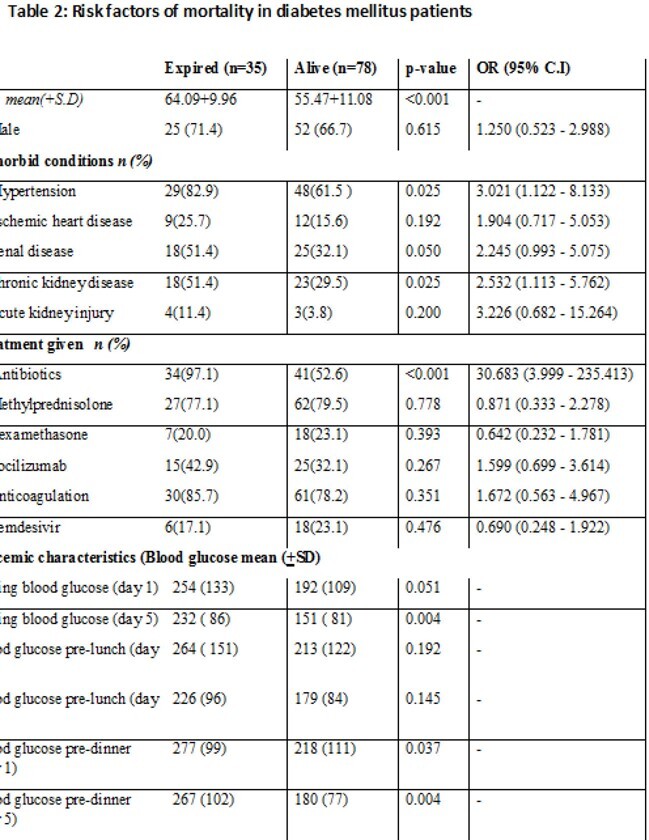

**Methods:**

This study is a single center retrospective observational review from Pakistan. Covid-19 diagnosed patients admitted during the months of March 2020 to March 2021were included. The patients were divided into DM and non-DM. Demographics, clinical variables and outcome were collected and compared between these groups. Glycemic control during hospitalization (blood sugar level fasting and pre-meal twice a day) was noted. Fasting glucose level >120 mg/dl, and random >200 mg/dl were considered as poor glycemic control. Survivors and non- survivors among DM patients were compared.

**Results:**

A total of 366 patients were included in this study, 113(30.87%) were DM and 253(69.12%) were non-DM. Mean age was comparable in two groups, however, patients in DM group were older (age >45 years, p=value 0.039). Significantly more patients with hypertension (p value< 0.001) and ischemic heart disease (p value< 0.001) developed Covid-19 in DM group. There was no difference in mortality among both groups (p=0.29). (Table 1) .In DM patients, the significant risk factors for mortality were age >60years, hypertension and renal disease. High fasting and pre-dinner blood glucose levels (mean) obtained at admission and day 5 were significantly associated with mortality. (Table2).

**Conclusion:**

Diabetic patients with advanced age and concomitant other comorbid conditions is associated with increased risk of death. In diabetic patients, more attention should be focus on dynamic monitoring and strict glycemic control as severe COVID 19 infection and its treatment with steroids can have a negative impact on outcome.

**Disclosures:**

**All Authors**: No reported disclosures.

